# Germline Variants in MLH1 and ATM Genes in a Young Patient with MSI-H in a Precancerous Colonic Lesion

**DOI:** 10.3390/ijms24065970

**Published:** 2023-03-22

**Authors:** Antonio Nolano, Giovanni Battista Rossi, Valentina D’Angelo, Raffaella Liccardo, Marina De Rosa, Paola Izzo, Francesca Duraturo

**Affiliations:** 1 Department of Molecular Medicine and Medical Biotechnologies and CEINGE Advanced Biotechnologies Scarl, “Francesco Salvatore” Napoli, University of Naples Federico II, 80131 Naples, Italy; 2 Endoscopy Unit, Istituto Nazionale Tumori–IRCCS–Fondazione G. Pascale, Via Mariano Semola, 80131 Naples, Italy

**Keywords:** ATM gene, Lynch syndrome, MMR genes, uncertain significance variants, synergist effect of risk alleles

## Abstract

Lynch syndrome (LS) is an autosomal dominant inherited disorder that primarily predisposes individuals to colorectal and endometrial cancer. It is associated with pathogenic variants in DNA mismatch repair (MMR) genes. In this study, we report the case of a 16-year-old boy who developed a precancerous colonic lesion and had a clinical suspicion of LS. The proband was found to have a somatic MSI-H status. Analysis of the coding sequences and flanking introns of the MLH1 and MSH2 genes by Sanger sequencing led to the identification of the variant of uncertain significance, namely, c.589-9_589-6delGTTT in the MLH1 gene. Further investigation revealed that this variant was likely pathogenetic. Subsequent next-generation sequencing panel analysis revealed the presence of two variants of uncertain significance in the ATM gene. We conclude that the phenotype of our index case is likely the result of a synergistic effect of these identified variants. Future studies will allow us to understand how risk alleles in different colorectal-cancer-prone genes interact with each other to increase an individual’s risk of developing cancer.

## 1. Introduction

Lynch syndrome (LS) is an autosomal dominant inherited disorder that mainly predisposes individuals to colorectal and endometrial cancer but is also associated with other extracolonic malignancies, such as stomach, small intestine, bladder, pancreatic, bile duct, and prostate cancer [1]. Germline pathogenic variants in DNA mismatch repair (*MMR*) genes such as *MLH1*, *MSH2*, *MSH6*, and *PMS2* are associated with a predisposition to this syndrome. However, few or no pathogenic variants in other *MMR* genes, such as *MSH3* and *MLH3*, have been associated with LS [2,3,4,5], although they have been reported to occasionally perform a cooperative role in cancer predisposition in combination with variants in the other MMR genes [6]. The inactivation of MMR genes results in the accumulation of mutations in microsatellite sequences (termed microsatellite instability (MSI)) found in coding and non-coding regions in cancer cells [2].

The identification of individuals who should undergo LS genetic testing is mainly based on personal and family history of cancer using the Amsterdam criteria or Bethesda guidelines [7,8] or through universal screening for LS based on MSI assessment or testing of MMR immunohistochemistry at the somatic level [9]. A diagnosis is finally confirmed by the identification of a germline pathogenic variant in one of the MMR genes.

Currently, rather than analyzing only the *MMR* genes associated with the syndrome, multigene panels are being analyzed to identify patients with a clinical suspicion of LS. Through these genetic panels, patients have been identified who did not have pathological variants in *MMR* genes but had them in other genes, such as mutations in biallelic *APC* or *MUTYH* or in genes not classically associated with colorectal cancer (CRC) risk such as *BRCA1/2* [10,11]. The ability to identify other genes as causative of LS may correlate with somatic differences in the colorectal carcinogenesis typical of LS.

The precursor lesion of an LS-related CRC is an adenomatous polyp, which is often proximal and may often be nonpolypoid rather than polypoid; moreover, it frequently displays villous features, high-grade dysplasia, and a preponderance of tumor-infiltrating lymphocytes [12]. In individuals with LS, adenomas may be associated with accelerated progression along the adenoma–carcinoma sequence.

Studies in the literature have reported three different patterns of colorectal carcinogenesis in Lynch patients, in which precancerous lesions can progress either toward an adenomatous phase or lead directly to an invasive tumor [13]. Lynch patients show an accelerated adenoma–carcinoma progression with an estimated interval of about 35 months compared to 10–15 years for sporadic tumors. Thus, colonoscopy appears to be the most effective form of prevention in these patients because it allows for the identification and removal of a preinvasive lesion and the early diagnosis of cancer in the absence of symptoms. In light of this, genetic tests assume considerable importance in the identification of individuals at risk of developing the CRC typical of LS patients.

In the first model, classical colorectal carcinogenesis follows the adenoma–carcinoma sequence, wherein MMR deficiency occurs after adenoma development [14]. Indeed, it has been described that 25% of adenomas retain the expression of MMR proteins [15]. In these cases, it has been shown that diffuse DNA methylation at CpG sites in promoter regions [16] could represent the “second hit” [17] that likely precedes MSI. Histologically, we note that polypoid precursor lesions are frequently found in the right colon [12,18,19] as well as the presence of differentiated mucinous cells associated with marked peritumoral lymphocytic inflammation [20]. In this type of colorectal carcinogenesis, the adenoma–carcinoma sequence is greatly accelerated [19,21], and somatic mutations in the CTNNB1 and APC genes develop before adenomas grow [22].

In the second model of colorectal carcinogenesis, the existence of colonic crypts showing mostly normal histological features but already lacking MMR protein expression have suggested that MMR-deficient CRC in LS patients could also be initiated therein [23]. Indeed, several recent studies have shown that approximately 75% of all adenomas in LS patients are MMR-deficient [22,23]. All tumors from LS patients exhibiting this type of colorectal carcinogenesis show MSI [24], which can inactivate tumor suppressor genes such as *TGFBR2* [15]. Mutations in *TGFBR2* have been found in 80% of early colorectal adenomas with high MSI [25]. The involvement of the WNT signaling pathway is associated with this second model of LS colorectal carcinogenesis. LS patients with the second type of colorectal carcinogenesis also show a high mutation rate in genes involved in DNA damage response signaling, such as *ARID1A*, *ATM*, and *BRCA2,* which play roles in homologous recombination and the repair of damage to double-stranded DNA in the colon [26].

Approximately 10% of LS-associated tumors show a third pattern of carcinogenesis in which they rapidly jump to an invasive phenotype resulting from colonic crypt foci that accumulate somatic mutations in genes such as *CTNNB1* and *TP53* [22]. It has been suggested that these mutations in *CTNNB1* and/or *TP53* may be involved in the malignant conversion of these foci [13,22,23,24,25,26,27].

Therefore, the identification of germline pathogenic variants in the other CRC-predisposing genes in patients with LS could act in a cooperative manner with respect to cancer predisposition and, moreover, explain the various models of colorectal carcinogenesis. This information could be used to program colonoscopies during the planned clinical surveillance of LS patients.

In this study, we report the case of a 16-year-old boy who developed a precancerous colonic lesion and had a clinical suspicion of LS.

## 2. Patient Report

**Figure 1:**
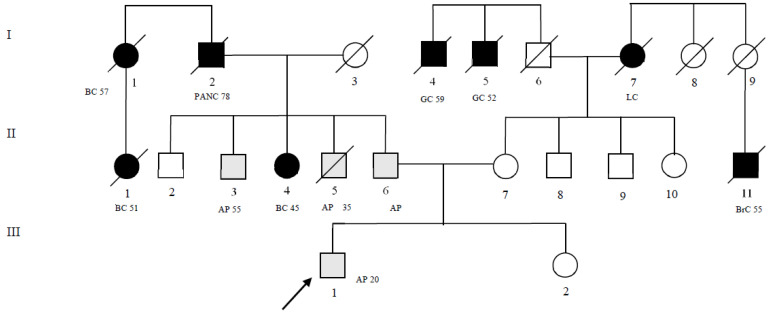
Family pedigree of the patient. The numbers next to each diagnosis denote the age at onset. Symbols and abbreviations used are denoted as follows: arrow, index case (proband); symbols with diagonal lines, succumbed; black symbol, CRC or tumors associated with Lynch syndrome; gray symbol, pre-cancerous lesions; CRC, colorectal cancer; LC, lung cancer; PANC, pancreatic cancer; BC, breast cancer; GC, gastric cancer; BrC, brain cancer; AP, adenomatous polyp; squares, males; circles, females. I–II–III generation.

Our proband performed a colonoscopy for rectal bleeding at age 16. Subsequently, an adenomatous rectal polyp was removed. The histological diagnosis was tubular adenoma with low-grade glandular dysplasia and the presence of chronic inflammatory lymphocytic infiltrates. Moreover, the proband’s family history was positive for adenomatous polyps and extracolonic cancers. A detailed pedigree is shown in Figure 1. A sample was collected from our patient after we were granted authorization from the Ethics Committee “Comitato etico per le attività Biomediche Carlo Romano” of the University of Naples Federico II (Naples, Italy), protocol no. 120/10. Once authorization was obtained, the study received ethical approval and written, informed consent was obtained from the patient.

## 3. Results

### 3.1. Analysis of MSI and Mutation Detection in MMR Genes

First, we analyzed MSI in our proband, detecting high MSI status (MSI-H) in the DNA extracted from tumor tissues and performing an analysis of the instability of three nucleotide markers (D5S346, BAT40, and BAT26). No V600E mutation was identified in the *BRAF* gene. Subsequently, all *MLH1* and *MSH2* exons were analyzed by Sanger sequencing. This sequencing analysis revealed an intronic variant: a deletion of the four intronic bases GTTT at the level of intron 7, upstream of exon 8, of the MLH1 gene; this mutation is named c.589-9_589-6delGTTT. This variant was already reported in the international database of the InSiGHT Group (http://www.insight-group.org/, accessed on 5 March 2023) and is classified as a class 3 mutation, that is, of uncertain pathogenetic significance. In our recent work, this variant was classified as a likely pathogenic variant via bioinformatic analysis [28]

### 3.2. Characterization of the c.589-9_589-6 GTTT Variant in the MLH1 Gene

**Figure 2:**
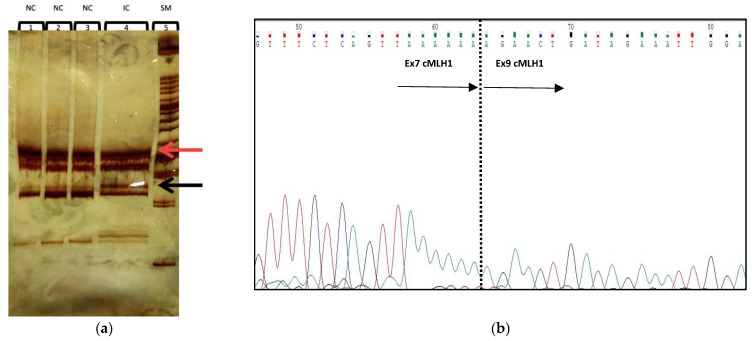
(**a**) Characterization of aberrant splicing fragment. Ten percent polyacrylamide gel electrophoresis of MLH1 cDNA RT-PCR analysis of fragment in a normal control (1, 2, and 3) and in the index case (4). Size marker XIV Roche (5). The black arrow indicates the wild-type band corresponding to the entire amplified fragment of 523 bp. The red arrow indicates a band with a lower molecular weight, which is not present in the negative controls. (**b**) MLH1 cDNA sequence analysis of abnormal band showing the skipping of exon 8. The dotted line shows skipping of entire exon 8 (c.589_c.677). NC, normal control; IC, index case; SM, size marker.

In silico analysis carried out using Human Splicing Finder (http://umd.be/Redirect.html/, accessed on 5 March 2023) software version 3.1 showed that this variant could lead to an alteration of the canonical splicing acceptor site with the activation of a new cryptic splicing site, as described by our team previously [28]. These computational data were confirmed by PCR analysis of the MLH1 cDNA fragment, including exons 6–10 of this gene. In particular, the band on which our analysis was concentrated has a lower molecular weight than the canonical one corresponding to the entire amplified fragment of 523 base pairs and was not present in the negative controls (Figure 2a). Each amplification product visualized on a 10% polyacrylamide gel was extracted and sequenced, as described in the Section 5. This band analysis revealed a splicing isoform of MLH1 mRNA caused by the skipping of exon 8 (c.589_c.677) (Figure 2b).

Thus, this variant was evaluated according to the criteria of the American College of Medical Genetics and Genomics (ACMG) [28,29]. Although this variant was already classified in our previously study as a variant likely pathogenic, in this study, we confirmed the prediction via computational analysis because this variant caused in vivo the skipping of exon 8, resulting in the formation of a truncated protein that was likely dysfunctional.

### 3.3. Next-Generation Sequencing Analysis

A germline DNA sample from the patient was tested with next-generation sequencing (NGS) analysis using the Hereditary Cancer Solution™ multigene panel (SOPHiA GENETICS, Lausanne, Switzerland). This genetic panel analyzes 26 genes involved in hereditary cancer syndromes. The analysis confirmed the presence of the c.589-9_589-6delGTTT variant in the *MLH1* gene and, moreover, identified two variants in the *ATM* gene, c.5975A>C p.(Lys1992Thr) and c.8734A>G p.(Arg2912Gly), both of which were classified by SOPHiA DDM software (which was updated to the latest reported version) as uncertain variants. These variants were also evaluated according to ACMG criteria and were classified as being of uncertain significance and probably pathogenic, respectively. No other pathogenic or VUS variants in these 26 genes were identified. Subsequently, other members of this family were analyzed by NGS analysis using a Hereditary Cancer Solution™ panel. In particular, individuals II-4, II-6, and II-7 (as well as that reported in Figure 1) showed the genotype reported in Table 1.

All of these variants identified by NGS analysis were confirmed by Sanger sequencing.

## 4. Discussion

LS is an autosomal dominant disease characterized by incomplete penetrance and variable expressivity in which a high variability of the risk of developing one of the tumors of the spectrum of the syndrome is also described in carriers of pathogenic variants in one of the *MMR* genes (*MLH1*, *MSH2*, *MSH6*, and *PMS2*) [1]. Therefore, an important aspect to consider in the molecular diagnosis of LS is the evaluation of the possible simultaneous presence of pathogenic variants in other genes beyond the aforementioned *MMR* when predicting the development of a hereditary tumor. Multiple germline variants in several genes predisposing colorectal cancers could confer a synergistic effect by increasing the individual’s risk of cancer development. Studies of this type could allow for a more precise assessment of the risk of cancer development in individuals with LS [6].

Therefore, in this study, we used a multigene panel marketed by SOPHiA GENETICS containing several genes predisposing one to the development of hereditary tumors. In addition to the genes responsible for LS, this panel includes, among others, the *ATM*, *PALB2*, *MRE11*, and *CHEK2* genes, which are expressed during the cell cycle and involved in DNA damage repair pathways [30,31]. Our index case is a boy who developed a precancerous lesion displaying an MSI-H phenotype and was found to be a carrier of a VUS in the *MLH1* gene. However, our analysis showed that this variant leads to the formation of abnormal mRNA that lacks the entire exon 8 and thus to a probably non-functional protein product, which is compatible with a defective repair system at the somatic level. Accordingly, reclassification with ACMG criteria [28,29] established that this VUS can be considered probably pathogenic and, therefore, likely to be responsible for the clinical phenotype of the index case. It is important to note that the MSI-H condition was found in DNA extracted from the precancerous lesion, an adenoma that the patient developed at a young age. Unfortunately, we were unable to perform an immunohistochemical analysis in order to verify the altered expression of the MLH1 protein. In agreement with data from the literature, about 75% of all adenomas of LS patients show MMR deficiency and follow the second pattern of colorectal carcinogenesis in which an early acquisition of a defective repair system precedes adenomatous formation [22,23]. These patients have been reported to somatically show a high mutation rate in genes involved in DNA damage response signaling, such as *ATM* and *BRCA2*. The NGS panel applied to our index case showed the presence of two other variants of uncertain pathogenetic significance in the other gene predisposing cancer development, the *ATM* gene: c.5975A>C and c.8734A>G. According to ACMG criteria, the c.8734A>G variant is classified as likely pathogenic because it falls in a region identified as a mutational hot spot, whereas the second variant, c.5975A>C, remains of uncertain significance. In light of these results, we hypothesized that the simultaneous presence of these two variants in the *ATM* gene with a variant in the *MLH1* gene may have been responsible for the very early onset of a precancerous colonic lesion in our index case. Cases of pathogenic germline variants simultaneously present in several genes involved in MMR and homologous recombination repair (HRR) have recently been described in the literature in several types of cancers [32]. Some of these cases have also been associated with the simultaneous loss of immune-expression in proteins of both MUTS and MUTL heterodimers (MLH1/PMS2 and MSH2/MSH6) in digestive system cancers [33].

The *ATM* gene encodes a protein kinase that plays an important role in activating cellular DNA repair responses. Recent studies have confirmed that some of the *ATM* gene variants are associated with an increased risk of breast cancer and susceptibility to CRC [34,35]. It is generally believed that germline *ATM* mutations lead to an increased risk of developing epithelial tumors such as those of the breast, stomach, and colon. Therefore, *ATM* is considered to be a moderate-risk gene, meaning that heterozygous *ATM* mutation carriers have a greater possibility of developing forms of tumor instability [28,35]. Furthermore, mutated *ATM* is believed to be involved in the alteration of Lynch phenotypic expression, and this gene is hypothesized to be particularly related to LS-related cancer manifestations [36].

Accordingly, the different variants identified could act cooperatively and be responsible for the carcinogenetic process described by the second model. Certainly, further molecular biology analyses are needed to verify this hypothesis. In the present work, we attempted to confirm the data through segregation analysis using the NGS SOPHiA genetic panel to analyze other family members. Unfortunately, one of the limitations of our study is undoubtedly the number of patients analyzed; indeed, few family members were willing to undergo the genetic test. Based on this analysis, we observed that the c.GTTT 589-9_589-6 variant in the *MLH1* gene and the c.5975A>C variant in the *ATM* gene were paternally derived. Both of these variants were identified in our proband’s paternal aunt (II-4), who developed breast cancer at 45 years old, and in the proband’s father (II-6), who reported the repeated development of precancerous lesions of the colon from the age of 38. The mother of our index case, who showed only the c.8734A>G variant in the *ATM* gene, is phenotypically healthy, and no other variants were identified in the 26 genes analyzed with the NGS panel. Therefore, the *ATM* variant c.8734A>G, which is considered by ACMG criteria to be probably pathogenetic because it is present in a gene with moderate penetrance for colon cancer, might not be sufficient to trigger the process of colorectal tumorigenesis if not present with another variant in a high-penetrance gene, as in the *MLH1* gene in this case.

We can conclude that the phenotype of our index case could be the result of a cumulative effect of all the identified variants. Accordingly, endoscopic surveillance for this proband has been scheduled every two years in accordance with the guidelines on endoscopic surveillance for subjects carrying possibly pathogenic variants in the *MMR* genes [37].

Future studies will allow us to understand how risk alleles in different CRC-predisposing genes may interact with each other to modify the phenotypic manifestation of LS and elucidate the variable expressivity typical of this syndrome. Our clinical case allows us to treat LS in relation to new diagnostic opportunities and thus obtain a better understanding of how gene variants can interact cooperatively in terms of predisposition to tumor development in order to plan the most appropriate clinical surveillance and achieve ever more personalized medicine.

## 5. Materials and Methods

Isolation of genomic DNA. Total genomic DNA was extracted from 4 mL peripheral blood lymphocytes using a BACC3 Nucleon kit (Amersham; GE Healthcare, Chicago, IL, USA). For each paraffin block, five 20 µm sections were cut and collected in a 1.5 mL microtube. Briefly, 1 mL of xylene was added to each tube followed by incubation at room temperature for 1 min to completely remove the paraffin. DNA was extracted after deparaffinization according to the protocol described by the manufacturer of the QIAamp DNA FFPE Tissue Kit (Qiagen, Hilden, Germany).

Evaluation of quantity and quality of extracted DNA. The quality of extracted DNA was evaluated using a NanoDrop OneC spectrophotometer (Fisher Thermo Scientific, Waltham, MA, USA) reading at 260 nm and ratios of 260/280 and 260/230 nm. Samples with the A260/A280 ratio falling within the range of 1.8–2.0 were considered to be of good quality. Subsequently, a Qubit 4 fluorometer (Invitrogen by Thermo Fisher Scientific, Waltham, MA, USA) allowed for the assessment of the dsDNA content in the isolated samples. DNA quality was evaluated by 1% agarose gel electrophoresis and visualized with ethidium bromide.

RNA extraction. Total RNA was extracted from the lymphocytes of the patient and three normal controls by using Trizol reagent (Invitrogen, Life Technologies, Carlsbad, CA, USA). A total of 1 microgram of RNA from each sample was retrotranscribed. cDNA was synthesized using 1.5 μg of total RNA, 500 ng of random hexamers, and 1 μL of Superscript III reverse transcriptase (Invitrogen, Life Technologies, CA, USA) in the presence of 4 μL 5X RT buffer, 1 μL DTT (0.1 M), and 1 mM dNTPs. The reaction was run for 50 min at 42 °C in a 20 μL reaction volume, heated to 70 °C for 15 min, and quick-chilled on ice. For MLH1 analysis, cDNA was amplified using the forward primer overlapping the junction between exons 6–7 (5′-AGTGGCTGGACAGAGGAAGA-3′) and the reverse primer located in exon 10 (5′-GATTTCTAAACTGAGGTACAGG-3′). The amplified fragment was visualized on 10 % polyacrylamide gel.

Microsatellite analysis and V600E BRAF mutation analysis. MSI was tested on paired samples of lymphocyte DNA and paraffin-embedded tumor tissues of the colon. The MSI status was evaluated with a fluorescent multiplex system comprising five mononucleotide repeats (BAT 25, BAT 26, NR 21, NR 24, and NR-27), three dinucleotide repeats (D2S123, D5S346, and D17S250), and two tetranucleotide repeats using the CC-MSI kit (AB ANALITICA, Padova, Italy) and subsequent capillary electrophoresis analysis using an ABI 3130xl Prism (Applied Biosystems, Thermo Fisher Scientific, Waltham, MA, USA). For V600E genotyping, genomic DNA extracted from paraffin-embedded tumor tissue and blood lymphocytes were amplified using customized primer pair (15F 5′ TGCTTGCTCTGATAGGAAAATGAGA 3′ and 15R 5′ GGCCCTGAGATGCTGCTGAG 3′) and sequenced in both the forward and reverse directions using an ABI 3100 Genetic Analyser (Applied Biosystems; Thermo Fisher Scientific, Waltham, MA, USA).

Variant analysis by Sanger sequencing. The coding regions corresponding to variants were amplified using customized primer sets (*MLH1* 8F:5′-ATGTTTCAGTCTCAGCCATGAG-3′; *MLH1* 8R: 5′-AGCCTGTGTATTTGACTAAAGC-3′; *ATM* 40F:5′- TTATTCTGTTTTGTTTGCCACCT-3′; *ATM* 40R:5′-TCAAGTCTGTCTACTCTAAGGCT-3′; *ATM* 60F:5′-ATGTGGTTTCTTGCCTTTGTAAA-3′; *ATM* 60R: 5′-ATCTCTAACCCGTGTGTGTG-3′). The PCR products were separated on a 1% agarose gel to check for unspecific amplicons. Subsequently, the PCR products were sequenced in both the forward and reverse directions using an ABI 3100 Genetic Analyzer (Applied Biosystems; Thermo Fisher Scientific, Waltham, MA, USA).

In silico analysis. We analyzed the novel variant detected in this study using the Human Splicing Finder (HSF) software (http://umd.be/Redirect.html/, accessed on 5 March 2023), a tool designed to predict the effects of mutations on splicing signals or identify splicing motifs in human sequences; it contains all available matrices for auxiliary sequence prediction and presents a novel position weight matrix to assess the strength of 5′ and 3′ splice sites and branch points.

NGS Library construction and Bioinformatics analysis. Patient DNA sample was examined using a capture-based target enrichment kit, Hereditary Cancer Solution™ (SOPHiA GENETICS, Switzerland), which targets the coding regions and sequence flanking regions (±25 bp) of 26 clinically relevant genes (target region of 105 kb) associated with breast and ovarian cancer and Lynch and intestinal polyposis syndromes (*ATM*, *APC*, *BARD1*, *BRCA1*, *BRCA2*, *BRIP1*, *CDH1*, *CHEK2*, *EPCAM*, *MLH1*, *MRE11A*, *MSH2*, *MSH6*, *MUTYH*, *NBN*, *PALB2*, *PIK3CA*, *PMS2*, *PMS2CL*, *PTEN*, *RAD50*, *RAD51C*, *RAD51D*, *STK11*, *TP53*, and *XRCC2*). Library preparation was performed using library construction protocol. The quality and quantity of the libraries obtained were assessed using a TapeStation 4200 System (Agilent Technologies, Santa Clara, CA, USA) with an Agilent DNA HS 1000 kit. Sequencing was performed on an Illumina MiSeq (Illumina, San Diego, CA, USA) with a V.2 Standard flow cell (500 cycles) reagent kit according to the manufacturer’s protocol. Variant calling was performed using the SOPHiA DDM^®^ platform (SOPHiA GENETICS, Switzerland). The average coverage for all 26 genes ranged from 488 to 5581×.

## Figures and Tables

**Table 1:** Genotypes of our index case’s relatives.

Patient Pedigree ID (Figure 1)	MLH1 Gene	ATM Gene
II-4	c.589-9_589-6delgttt	c.5975A>C
II-6	c.589-9_589-6delgttt	c.5975A>C
II-7	-	c.8734A>G
